# Region-specific elevations of glutamate + glutamine correlate with the sensory symptoms of autism spectrum disorders

**DOI:** 10.1038/s41398-021-01525-1

**Published:** 2021-07-29

**Authors:** Jason L. He, Georg Oeltzschner, Mark Mikkelsen, Alyssa Deronda, Ashley D. Harris, Deana Crocetti, Ericka L. Wodka, Stewart H. Mostofsky, Richard A. E. Edden, Nicolaas A. J. Puts

**Affiliations:** 1 Russell H. Morgan Department of Radiology and Radiological Science, The Johns Hopkins University School of Medicine, Baltimore, MD USA; 2 F. M. Kirby Research Center for Functional Brain Imaging, Kennedy Krieger Institute, Baltimore, MD USA; 3 Department of Forensic and Neurodevelopmental Sciences, Sackler Institute for Translational Neurodevelopment, Institute of Psychiatry, Psychology, and Neuroscience, King’s College London, London, UK; 4 Center for Neurodevelopmental and Imaging Research, Kennedy Krieger Institute, Baltimore, MD USA; 5 Department of Radiology, University of Calgary, Calgary, Canada; 6 Center for Autism and Related Disorders, Kennedy Krieger Institute, Baltimore, MD USA; 7 Department of Neurology, The Johns Hopkins University School of Medicine, Baltimore, MD USA; 8 Department of Psychiatry and Behavioral Sciences, The Johns Hopkins University School of Medicine, Baltimore, MD USA; 9 MRC Centre for Neurodevelopmental Disorders, King’s College London, London, UK

**Keywords:** Molecular neuroscience, Diagnostic markers

## Abstract

Individuals on the autism spectrum are often reported as being hyper- and/or hyporeactive to sensory input. These sensory symptoms were one of the key observations that led to the development of the altered excitation-inhibition (E-I) model of autism, which posits that an increase ratio of excitatory to inhibitory signaling may explain certain phenotypical expressions of autism spectrum disorders (ASD). While there has been strong support for the altered E-I model of autism, much of the evidence has come from animal models. With regard to in-vivo human studies, evidence for altered E-I balance in ASD come from studies adopting magnetic resonance spectroscopy (MRS). Spectral-edited MRS can be used to provide measures of the levels of GABA + (GABA + macromolecules) and Glx (glutamate + glutamine) in specific brain regions as proxy markers of inhibition and excitation respectively. In the current study, we found region-specific elevations of Glx in the primary sensorimotor cortex (SM1) in ASD. There were no group differences of GABA+ in either the SM1 or thalamus. Higher levels of Glx were associated with more parent reported difficulties of sensory hyper- and hyporeactivity, as well as reduced feed-forward inhibition during tactile perception in children with ASD. Critically, the finding of elevated Glx provides strong empirical support for increased excitation in ASD. Our results also provide a clear link between Glx and the sensory symptoms of ASD at both behavioral and perceptual levels.

## Introduction

While the phenotypical expression of autism spectrum disorders (ASD) is heterogeneous, ~95% of individuals with ASD present as being either hyper- and/or hyporeactive to sensory stimuli [1–4]. Since sensory symptoms can precede the development or presentation of what are considered to be the hallmark characteristics of ASD (i.e., deficits of social communication and restricted, repetitive behaviors) [5], it has been suggested that the pathophysiology underlying these sensory abnormalities may actually drive the development of the other core symptoms of autism [6], though the evidence in support of this suggestion is still being developed and debated. Given the proposed importance of sensory processing to neurodevelopment and functioning in ASD, many studies conducted over the last two decades attempt to both characterize the nature of sensory symptoms of ASD [7–9] and to understand their underlying neurobiological causes [10, 11].

Investigations of sensory reactivity, or how someone responds to sensory stimuli, have principally relied on the use of self, parent, and/or teacher reports to describe how children with ASD react to a range of stimuli across different sensory domains and contexts (e.g., ‘how does your child react to loud noises in their environment?’ or ‘how does your child feel about wearing certain articles of clothing?’) [2, 12–14]. Studies leveraging self, parent, and/or teacher questionnaires to characterize sensory symptoms in ASD have generally found that when compared to children with other neurodevelopmental disorders, as well as typically developing controls (TDC), children with ASD are far more likely to present with symptoms of both hyperreactivity and hyporeactivity, with the evidence for the presence of the latter being more heterogeneous [15]. Interestingly, individuals with ASD who are either hyper- and/or hyporeactive to stimuli in one sensory domain also tend to be similarly hyper- and/or hyporeactive to stimuli in other domains (e.g., a child who is hyperreactive to auditory stimuli also tends to be hyper-reactive to tactile stimuli), suggesting these symptoms to be domain-general rather than domain-specific [16].

Unlike sensory reactivity, which relies on subjective reporting, sensory sensitivity can be assessed objectively using psychophysical methods [17]. Psychophysical paradigms can be used to assess low-level sensitivity (i.e., detection and discrimination) across various perceptual domains [17]. Performance on these paradigms can be typically linked to known neurophysiological processes [18], making inferences from altered sensitivity to abnormal neurophysiological functioning possible. Through psychophysics, we and others have observed patterns of altered sensory sensitivity in ASD that were broadly consistent with reduced or inefficient inhibitory signaling [19–28]. For instance, our own work conducted in the tactile domain revealed that children with ASD showed less of an increase in detection thresholds when perceiving stimuli of increasing intensity and frequency. Indeed, this pattern is consistent with reduced thalamocortical feed-forward inhibition and altered GABAergic signaling [29–32]. Further, we recently found that children with ASD, as opposed to children with attention-deficit hyperactivity disorder (ADHD), have particular difficulties with discriminating between the amplitude and frequency of sequentially or simultaneously delivered tactile stimuli [16]. Likewise, these difficulties point to altered lateral inhibition, a process that helps sharpen receptive fields for enhanced discrimination, and as with feed-forward inhibition, is also principally dependent on GABA. Importantly, our findings in the tactile domain parallel similar findings in both auditory [33] and visual [34] domains, again highlighting the domain-generality of sensory symptoms in ASD.

When taken together, the evidence from investigations of both sensory reactivity and perceptual sensitivity in ASD are beginning to suggest that being hyper- and hyporeactive to sensory input originates from alterations at the perceptual level. More critically, since both feed-forward inhibition and lateral inhibition are dependent on GABAergic processes [35–38], results from these psychophysical experiments further suggest that altered sensory perception in ASD is, at least in part, due to reduced or inefficient GABAergic functioning in ASD. Indeed, the idea of abnormal GABA in ASD is not novel and there are many other lines of supporting evidence [39]. Since its original proposal by Hussman [40], and the proliferation of the idea through the frequently cited review article by Rubenstein and Merzenich [41], studies conducted from various levels of analysis have found support for the altered excitation-inhibition (E-I) balance theory of ASD, which suggests that the symptoms of autism can be explained by a shift in the overall balance of glutamatergic ‘excitatory’ and GABAergic ‘inhibitory’ signaling [42].

The role of E-I balance in ASD has been investigated in both humans and in animal models [43]. While animal-based work can provide important insights into the pathophysiology of E-I balance in autism, due to the complexity of behavioral symptom presentation of ASD, animal-based work cannot capture the associations between neurobiological alterations and behavior as well as human studies. Magnetic resonance spectroscopy (MRS) is a non-invasive technique that allows for the measurement of neurometabolite levels in-vivo in both humans and animals [44]. Edited MRS can be used to measure the levels of neurometabolites that would otherwise be difficult to detect at low field strengths [45]. Of relevance to the current study, edited MRS can be used to measure the levels of GABA and glutamate (note that GABA measures from spectral editing are contaminated by overlapping macromolecular signals and glutamate is difficult to resolve from the similar glutamine, hence GABA and glutamate levels acquired by MRS are typically referred to as GABA+ and Glx [glutamate + glutamine], respectively [46]).

Findings from studies applying edited-MRS to assess GABA+ and glutamate in ASD have been rather inconsistent [47], perhaps owing to the heterogeneity of the condition itself, but also to the variety of MRS methods, brain regions, and age cohorts represented across these studies [48]. In the current study, we focused on Glx and GABA+ levels, interpreting them as broad surrogate markers of excitation and inhibition in regions specifically involved in tactile processing: the primary sensorimotor cortex (SM1) [49–51] and the thalamus (Thal) [52–54]. First, we compared relative concentrations of GABA+ and Glx in SM1 and Thal between ASD and controls, hypothesizing that GABA+ would be decreased in ASD (as in our prior work [30]), and making no specific directional hypothesis regarding Glx. We then conducted exploratory analyses to investigate the associations between Glx and GABA+ to parent reports of sensory reactivity and psychophysically derived measures of tactile sensitivity. With these exploratory analyses, we broadly hypothesized that lower GABA+ and higher Glx levels would be associated with both greater sensory hyper- and hyporeactivity and lower tactile sensitivity (e.g., higher perceptual thresholds and reduced feedforward inhibition).

## Materials and methods

### Enrollment and clinical assessment

In all, 73 children with ASD and 92 TDCs were enrolled to participate in this study (see Table 1 for demographics). A parent of each child assented to testing and provided written informed consent to their child participating. The study protocol was approved by the local institutional review boards of the Kennedy Krieger Institute and The Johns Hopkins University School of Medicine.

**Table 1:** Descriptive statistics for demographic variables and key clinical outcomes.

	ASD			TDC			
	*N*	*M*	SD	*N*	*M*	SD	*p*
Demographics							
Age	44	10.32	1.49	62	9.69	1.21	0.019
Sex M (F)	42 (2)	–	–	46 (16)	–	–	0.009
Handedness R (L)	40 (4)	–	–	57 (5)	–	–	>0.999
WISC4 - FSIQ	3	102.33	4.04	13	114.69	7.59	0.017
WISC5 - FSIQ	41	101.49	17.91	49	112.94	11.19	<0.001
ADOS							
Social interaction	44	7.36	3.58	61	–	–	–
Communication	44	3.25	1.91	61	–	–	–
Stereotyped	44	2.7	1.82	61	–	–	–
Total	44	13.32	6.6	61	–	–	–
Sensory Experience Questionnaire							
SEQ hyperreactivity	37	2.79	0.57	40	1.39	0.22	<0.001
SEQ hyporeactivity	37	2.02	0.52	40	1.21	0.24	<0.001
SEQ sensory seeking	37	2.47	0.6	40	1.45	0.4	<0.001
MRS metabolite levels (residuals after modeling effects of acquisition phase)							
* SM1*							
Glx/Cr	44	0.00556	0.01319	59	−0.00415	0.01815	–
Glx (IU)	44	0.68312	1.59195	59	−0.50944	1.68914	–
GABA+/Cr	42	0.00022	0.01226	53	−0.00018	0.01056	–
GABA+(IU)	41	0.03637	0.33894	54	−0.02761	0.31498	–
* Thal*							
Glx/Cr	20	0.00049	0.00524	26	−0.00037	0.00662	–
Glx (IU)	18	0.0095	0.69202	29	−0.0059	0.76507	–
GABA+/Cr	24	−0.00005	0.0138	31	0.00004	0.01342	–
GABA+(IU)	26	0.09175	0.63336	31	−0.07695	0.51141	–

MRS descriptive statistics are referring to the mean (M) and standard deviation (SD) of the residuals from the models used to combine across acquisition phases. Note: categorical variables (sex and handedness) were compared using *χ*^2^ test of independence. All other variables were compared using independent samples t-tests. “−” – MRS descriptive statistics are not compared here, see main text.

N = Number of participants, *M* = Mean, SD = Standard Deviation, WISC – Wechsler Intelligence Scale – Children, FSIQ = Full Scale IQ, ADOS = Autism Diagnostic Observation Scale, ADI = Autism Diagnostic Interview; SEQ = Sensory Experience Questionnaire, SM1 = sensorimotor cortex, Thal = thalamus, Glx = glutamate + glutamine, Cr = creatine, IU = institutional units.

Participants with ASD met diagnostic criteria based on the Diagnostic and Statistical Manual of Mental Disorders Fifth Edition (DSM‐5) criteria [55] and was verified using the Autism Diagnostic Observation Schedule Second Edition (ADOS-2) [56] and Autism Diagnostic Interview‐Revised (ADI-R) [57]. The Wechsler Intelligence Scale for Children ‐ Fourth (WISC-IV) [58] and Fifth (WISC-V) [59] editions were used to determine general cognitive and intellectual ability. Handedness was determined using the Edinburgh Handedness Inventory [60].

Children with identifiable genetic causes of autism (e.g., Fragile X syndrome) and other neurological disorders were excluded. Children with full‐scale IQ scores below 80 were excluded from participation unless there was a 12-point or greater index discrepancy, in which case either the Verbal Comprehension Index or perceptual reasoning index (PRI) was required to be ≥80 and the lower of the two was required to be ≥65.

Participants in the ASD cohort were instructed to discontinue stimulant medication on the day of participation, as well as the day before, but were allowed to take other psychotropic medications requiring extended washout. None of the children in the TDC cohort were prescribed psychoactive medications.

### MRS of GABA+ and Glx

#### Acquisition

All structural MRI and MRS data were acquired on a Philips 3T MRI scanner (Philips Healthcare, Best, The Netherlands). During the data collection period, the MRI scanner underwent hardware and software upgrades. Our methodological research in the field of edited MRS also saw improvements in the acquisition parameters of the protocol initially selected for acquiring GABA+ and Glx. Thus, data were collected in three separate acquisition phases (the parameters of which are described in detail in the Supplementary Methods).

**Fig. 1:**
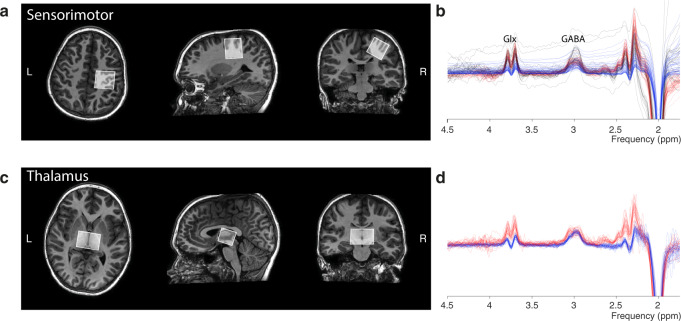
Voxel placement and example spectra. **a** Depiction of voxel placement for the left primary sensorimotor cortex (SM1) in a randomly selected participant. **b** Spectra from all the participants, color coded by the acquisition phase from which they were collected (black = phase 1, blue = phase 2, and red = phase 3 – see Supplementary Methods). **c** Voxel placement for the thalamus (Thal) in a randomly selected participant. **d** Spectra from all participants, color coded by acquisition phase. Note: no phase 1 data were acquired in the Thal.

Following a fast survey image, a high-resolution (1 mm^3^ isotropic) T_1_-weighted (MP-RAGE) image was acquired to guide voxel placement and to be used for tissue segmentation in subsequent data processing. In all phases (labeled 1 to 3), an isotropic MRS voxel (30 × 30 × 30 mm^3^ [27 ml]) was placed in the right primary sensorimotor cortex (SM1). The voxel was centered using the hand-knob in the central sulcus as a guiding anatomical landmark [61] and was rotated to be aligned with the dorsolateral surface (see Fig. 1a). For phases 2 and 3, an additional MRS voxel (26 mm (AP) × 24 mm (CC) × 40 mm (LR) [25 ml]) was placed in the thalamus (Thal). This voxel was positioned so as to include both halves of the thalamus, sacrificing information on laterality to achieve sufficient signal-to-noise ratio (Fig. 1b). Common parameters across all phases were: 320 averages per voxel; TR/TE = 2000/80 ms; 2048 samples; 2 kHz spectral width; and VAPOR water suppression [62].

#### Preprocessing and quantification

A comprehensive description of how data were preprocessed and quantified is provided in Supplementary Methods. Briefly, metabolite concentrations were estimated using Gannet (version 3.1), a software package developed for the automated batch processing of edited MRS data [63]. Using tissue segmentation implemented in SPM12 [64], the fractional tissue volumes for gray matter (GM), white matter (WM), and cerebrospinal fluid (CSF) were determined for each voxel of the T_1_-weighted image acquired at the start of the scan session. GABA+ and Glx measurements were then corrected for interindividual differences in voxel compositions of GM, WM, and CSF to increase precision and accuracy of quantification [65, 66]. Metabolite estimates are typically referenced to either tissue water or total creatine [45]. To account for the possibility that our results were driven by interindividual differences in the reference (i.e., either tissue water or total creatine) rather than our metabolites of interest, GABA+ and Glx estimates were quantified relative to both water (alpha-tissue corrected, referred to as GABA + (IU) and Glx (IU)) and creatine (referred to as GABA + /Cr and Glx/Cr).

Data quality was visually inspected by GO (~9 years of experience using edited MRS). Individual fits were excluded from analyses if either the data themselves or the fits were considered unusable (e.g., due to excessive subject motion), resulting in strongly diminished spectral quality (e.g., subtraction artefacts, or a degree of lipid contamination in cortical voxels strong enough to interfere with the peak modeling). Finally, estimates across the acquisition phases were compared (see Supplementary Table 1) and combined using linear mixed-effect models treating acquisition phase as a random factor. The residual values for each participant (i.e., the variance not accounted for by acquisition phase) were then used as estimates of GABA+ and Glx. For reasons of readability, we continue to refer to these variables as GABA + (IU), Glx (IU), GABA+/Cr, and Glx/Cr rather than referring to them as residuals (e.g., residual GABA + (IU)).

### Sensory reactivity and sensitivity

#### Sensory Experience Questionnaire

Sensory reactivity was assessed using the Sensory Experience Questionnaire (SEQ) [2]. The SEQ is intended to be completed by parents to assess how their child reacts to everyday sensory experiences across different sensory domains. The SEQ was specifically designed to characterize sensory features in young children with ASD and discriminates between patterns of hyper- and hyporeactivity, as well as sensory seeking behaviors (described as craving of certain sensory stimuli, perhaps as a way to self-regulate stimulation levels [67]) among children with ASD, children with other developmental disorders and TDCs. Questions such as ‘how often does your child react sensitively to unexpected or loud sounds?’ or ‘how often does your child seem slow to look at objects that are placed or held near him/her’ are used to probe sensory hyper- and hyporeactivity respectively. Similarly, questions such as how often does your child: ‘smell objects or toys during play or other activities’ are used to probe sensory seeking behaviors. Total item scores from the SEQ were used for subsequent analysis.

#### Tactile sensitivity

**Fig. 2:**
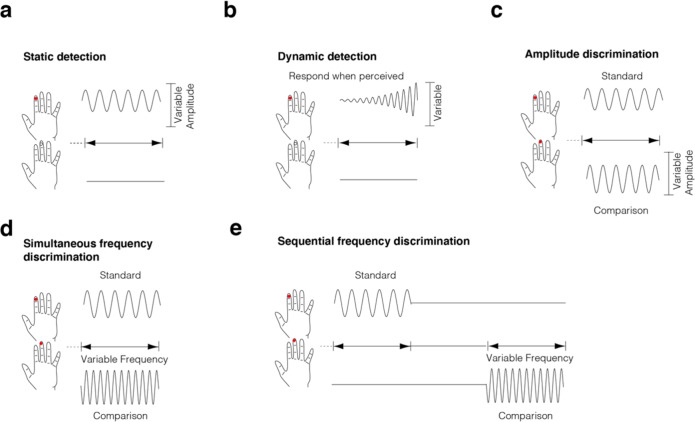
Visual schematic of relevant tasks from the vibrotactile battery. **a** Static detection protocol in which participants must detect which finger (left digit 1 or left digit 2) received the stimulation. Stimuli are presented with a fixed frequency and an amplitude that is increased or decreased based on incorrect and correct responses respectively. **b** Dynamic detection protocol in which participants are instructed to respond immediately after they perceive a stimulus of increasing amplitude. **c** Amplitude discrimination protocol in which a standard stimulus with a fixed amplitude and frequency is compared to a comparison stimulus in which the amplitude varies based on performance (i.e., the difference in amplitudes between the standard and comparison stimulus is increased following an incorrect response but decreases following an incorrect response). **d** Simultaneous frequency discrimination, similar to the amplitude discrimination protocol but rather than the stimulus amplitude changing based on performance, frequency of the comparison stimulus changes instead. **e** Sequential frequency discrimination, like the simultaneous frequency discrimination but stimuli are delivered sequentially (requiring less lateral inhibition and likely requires more accurate temporary storage of stimulus characteristic for later comparison). Full details about the parameters and timing of the stimuli are provided in Supplementary Methods.

Perceptual sensitivity was assessed in the tactile domain using a battery of vibrotactile tasks (total duration ~40 mins) [68]. Participants were instructed to rest their left hand on a CM4 four-digit tactile stimulator (Cortical Metrics, Carrboro, NC). The stimulator was used to deliver vibrotactile stimuli within the flutter range (25–50 Hz) to the glabrous skin on digit 2 (LD2 - index finger) and digit 3 (LD3 - middle finger) of the left hand via two cylindrical plastic probes with 5 mm diameter. The battery consisted of 11 tasks, grouped into five domains (reaction time, detection threshold, amplitude discrimination, frequency discrimination, and temporal order judgment), with three conditions in the amplitude discrimination domain and two conditions for each of the other domains. For brevity, we focus on the tasks that have previously revealed group differences between ASD and TDCs [16, 19], namely the static and dynamic detection, and amplitude and frequency discrimination paradigms. See Fig. 2 and Supplementary Methods for further details.

### Statistical analysis

All statistical analyses were conducted using the R programming language (version 4.0.2) in R Studio (version 1.2.1335) [69]. The code used to generate the results and figures of this manuscript are available online through the Open Science Framework (https://osf.io/xhqwu/). Alpha levels for all analyses were set to 0.05. Where relevant, partial eta-squared (*η*_p_^2^) was estimated using the ‘effectsize’ package [70]. Bayes factors were estimated using the ‘BayesFactor’ package [71] using non-informative Jeffreys’s priors [72]. Statistical outliers for all relevant variables were removed using the median absolute deviation method using a 2.5 standard deviation threshold [73].

Quantitative MRS data quality was first assessed using independent samples *t*-tests comparing fit values and full-width half maximum (FWHM) of the modeled NAA signal in the non-edited spectrum for each acquisition phase (see Supplementary Table 2). To determine whether there were group differences in GABA + and Glx levels for both regions of interest, a 2 × 2 repeated-measures ANOVA was conducted to compare GABA + and Glx levels between Region (SM1 and Thal) and Group (ASD and TDC). To determine whether interindividual differences in GABA+ and Glx in both SM1 and Thal were significantly related to sensory reactivity, we conducted Pearson’s correlation analyses between GABA+ and Glx from SM1 and Thal to total sensory hyper- and hyporeactivity, and sensory seeking scores from the SEQ. Similarly, to determine whether neurometabolite levels in our regions of interest were significantly related to sensory sensitivity, we conducted Pearson’s correlation analyses between GABA + and Glx of SM1 and Thal to perceptual thresholds measured through the vibrotactile psychophysical battery. All correlations were first conducted across the entire sample (i.e., considering ASD and TDC participants together) before considering whether there was a moderating effect of Group.

Additional exploratory analyses required for interpretation of our results are described in-line below. The sample size presented here is larger than many existing studies (including our own work) which have identified group differences or correlations using MRS-derived measures of excitation and inhibition. Bonferroni’s method was applied to analyses that were (a) conducted following a significant interaction effect (each *p*-value was multiplied by the number of slopes analyzed), or (b) involved multiple correlation analyses between a given metabolite and multiple scale items on a questionnaire (each *p*-value was multiplied by the number of associations conducted between each metabolite and each item of each questionnaire). Alpha was set 0.05 and all tests conducted were two-sided.

## Results

Descriptive statistics for all relevant variables in the subsequent analyses below are presented in Table 1. Descriptive statistics for performance on the tactile protocols are presented in Supplementary Table 3. Results for the analyses conducted on Glx and GABA+ referenced to creatine are presented in Supplementary Results. All instances of *M* and SD presented in-text refer to mean and standard deviations.

### Comparing metabolite levels of SM1 and Thal between ASD and TDCs

#### Elevated levels of Glx in SM1 but not Thal in ASD

**Fig. 3:**
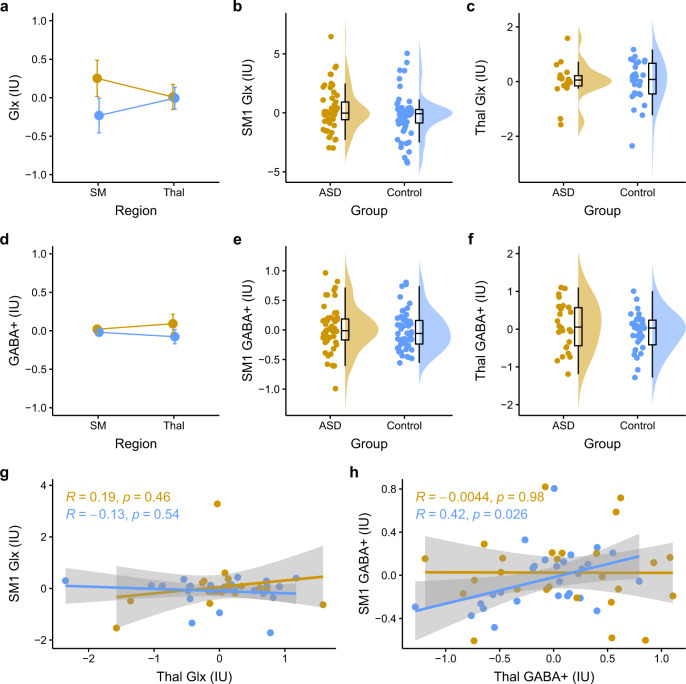
Region-specific increase in Glx but not GABA+ in ASD. The ASD and TDC group are depicted as yellow and blue (respectively). **a** Evidence towards a Group by Region interaction effect on Glx (IU). **b** Higher Glx (IU) levels in the SM1 voxel for children in the ASD compared to TDC group. **c** Low evidence towards a meaningful group difference in Glx (IU) levels in the Thal voxel**. d** Lack of evidence towards a significant Group by Region interaction effect on GABA + (IU) levels. **e** Comparable GABA + (IU) in SM and **f** Thal voxels between children in the ASD and TDC groups. **g** Linear relationship between SM Glx and Thal Glx in ASD that is otherwise absent in TDC. **h** No relationship between SM GABA+ and Thal GABA+ in either ASD or TDC. IU institutional units, Glx glutamate + glutamine, Cr creatine, SM1 primary sensorimotor cortex, Thal thalamus, ASD autism spectrum disorders, TDC typically developing controls. Note: *p*-values presented on panels **g** and **h** are not corrected for multiple comparisons, please refer to the *p*-values in the main text. Error bars in panels **a** and **d** represent standard error. The shaded area around the line of best fit represents the 95% confidence interval.

While there was very strong evidence for a significant main effect of Group on Glx (IU) levels [*F*(1, 146) = 12.27, *p* < 0.001; *η*_p_^2^ = 0.078; BF_10_ = 38.16], there was also substantial evidence for a Group by Region interaction effect [*F*(1, 146) = 5.22, *p* = 0.024; *η*_p_^2^ = 0.034; BF_10_ = 7.67] (Fig. 3a). Subsequent simple main effect analyses found strong evidence for an effect of Group on SM1 Glx (IU) [*F*(1,101) = 13.19, *p*_Bonferroni_ < 0.001; *η*_p_^2^ = 0.116; BF_10_ = 59.48], which was absent for Thal [*F*(1, 45) = 0.00, *p*_Bonferroni_ = 0.999; *η*_p_^2^ = 0.000; BF_10_ = 0.30]. Indeed, although children in the ASD (*M* = 0.68, SD = 1.59) group had higher SM1 Glx levels compared to the TDC group (*M* = −0.01, SD = 0.69), the groups had otherwise comparable Thal Glx levels [ASD: *M* = 0.01, SD = 0.69; TDC: −0.01, SD = 0.77] (see Fig. 3b, c). A similar pattern of results was observed when Glx was referenced to creatine (see Supplementary Results and Supplementary Fig. 1), suggesting that the pattern of these effects was not driven by the reference signal.

#### Comparable levels of GABA+ in SM1 and Thal between ASD and TDCs

In contrast to Glx, there was evidence against a main effect of both Group [*F*(1,148) = 2.14, *p* = 0.146, *η*_p_^2^ = 0.014; BF_10_ = 0.47] and Region [*F*(1, 148) = 0.00, *p* = 0.972; *η*_p_^2^ = 0.022; BF_10_ = 0.18] on GABA + levels. There was also evidence against an interaction effect [*F*(1, 148) = 0.52, *p* = 0.474; *η*_p_^2^ = 0.003; BF_10_ = 0.08] (Fig. 3d). Indeed, SM1 GABA+ levels were comparable between ASD and TDC groups for SM1 [(ASD: *M* = 0.04, SD = 0.34; TDC: *M* = −0.03, SD = 0.31)] and Thal [(ASD: *M* = 0.09, SD = 0.63; TDC: *M* = −0.08, SD = 0.51)] (Fig. 3e, f). As with Glx, the same pattern of results was observed for GABA + /Cr.

#### Between-region correlation of metabolites: evidence for increased glutamatergic thalamocortical connectivity in ASD

Altered thalamocortical connectivity in ASD has long been suggested [74–76]. While MRS can only provide region-specific estimates of neurometabolite levels, it is possible to assess how neurometabolites levels are associated across regions, as well as whether and how those associations differ between groups. Given that thalamocortical relay neurons are predominantly glutamatergic [77], correlations between Glx levels of SM1 and Thal could be associated with thalamocortical connectivity.

As an indirect assessment of thalamocortical connectivity, we compared the correlation between SM1 Glx and Thal Glx between ASD and controls. While there was a significant positive correlation between SM1 Glx and Thal Glx across the whole sample [(*r* = 0.42, *F*(1, 54) = 11.45, *p* = 0.001; *η*_p_^2^ = 0.18; BF_10_ = 28.70)], there was very strong evidence towards a moderating effect of Group [(*F*(1, 52) = 5.72, *p* = 0.006); *η*_p_^2^ = 0.08; BF_10_ = 44.34]. Subsequent simple slope analyses revealed that while there was no meaningful association between SM1 Glx and Thal Glx in the TDC group (*r* = 0.01, *p*_Bonferroni_ = 0.999), there was a moderate to strong positive correlation in the ASD group (*r* = 0.55, *p*_Bonferroni_ = 0.007) (Fig. 3g). These results provide indirect support for increased thalamocortical connectivity in ASD compared to controls. For completion, we also assessed inter-region associations of GABA+ . There was no correlation between SM1 GABA+ and Thal GABA+ [(*r* = −0.15, *F*(1, 52) = 1.15, *p* = 0.288; *η*_p_^2^ = 0.02; BF_10_ = 0.44)]. There was also no moderating effect of Group on the associations between SM1 and Thal GABA+ [*F*(1, 51) = 0.68, *p* = 0.415; *η*_p_^2^ = 0.01; BF_10_ = 0.16)]. See Fig. 3h. The same pattern of effects were observed for Glx/Cr and GABA+/Cr (see Supplementary Fig. 2).

### Correlations between metabolite levels of SM1 and Thal with sensory reactivity

#### SM1 Glx is associated with both hyper- and hyporeactivity

**Fig. 4:**
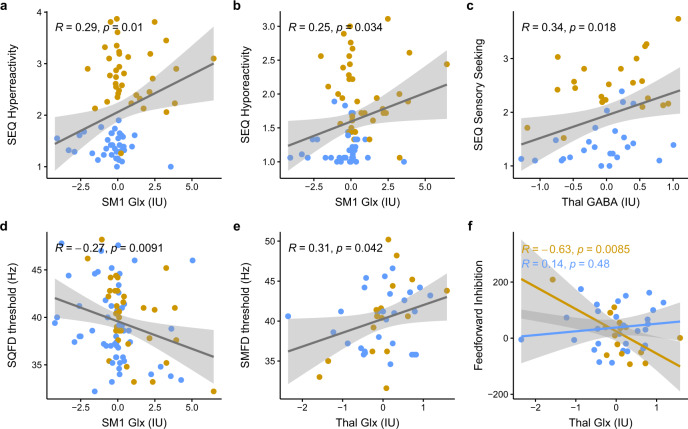
Associations between Glx and GABA+ of SM1 and Thal with sensory reactivity and tactile sensitivity. The ASD and TDC group are depicted as yellow and blue (respectively). Positive correlations were identified between Glx (IU) levels of SM1 and **a** Hyper- and **b** hyporeactivity scores on the SEQ. **c** Thal GABA (IU) levels also positively correlated with sensory seeking scores. **d** SM1 Glx (IU) levels showed evidence of a negative association to SQFD thresholds and **e** Thal Glx (IU) levels showed evidence of a positive association to SMFD thresholds. While we note that the associations presented in panel **d** and **e** do not survive correction for multiple comparisons, the associations were in the same direction when Glx was referenced to creatine, and the association in panel e was also stronger (*r* = 0.41, *p* = 0.0062), suggesting that these associations were neither simply due to the reference variable or spurious. See Supplementary Results and Supplementary Fig. 2. **f** There was a strong negative association between Thal Glx (IU) levels and feedforward inhibition in the ASD group that was otherwise absent in the control group. Indeed, a similar pattern of effect (i.e., an interaction effect showing a negative association between Thal Glx and feedforward inhibition in the ASD group that is otherwise absent in the TDC group) was also identified when Glx was referenced to creatine. See Supplementary Results – Fig. 2f. IU institutional units, Glx glutamate + glutamine, Cr creatine, SM1 primary sensorimotor cortex, Thal thalamus, ASD autism spectrum disorders, TDC typically developing controls. Note: although the correlations in panels **a**–**d** are driven by extreme values on either end, the variables are approximately normally distributed at the whole sample level. This suggests that these measures are accurately representing the continuum of possible scores on each of the measures. Note: *p*-values presented on all panels are not corrected for multiple comparisons, please refer to the *p*-values in the main text. The shaded area around the line of best fit represents the 95% confidence interval.

There were significant associations between SM1 Glx and parent reported hyper- (*r* = 0.36, *p*_Bonferroni_ = 0.005; Fig. 4a) and hyporeactivity (*r*_Bonferroni_ = 0.32, *p*_Bonferroni_ = 0.015; Fig. 4b) on the SEQ. There was no meaningful association between SM1 Glx and sensory seeking (*r* = 0.11, *p*_Bonferroni_ = 0.999). There were no moderating effects of Group for any of these associations (all *p*_Bonferroni_ > 0.999). We recognize that the associations between SM1 Glx with hyper- and hyporeactivity can be interpreted as being driven by clustering within the groups. Typically, when data is clustered within groups of a correlation, it is argued that the correlation is being driven by group differences, rather than reflecting a true correlation. Indeed, given the group differences on both SEQ hyper- (*p* < 0.001) and hyporeactivity (*p* < 0.001), as well as SM1 Glx (see Fig. 3b), the data points are clustered in such a way that the children in the ASD group are clustered towards the top right and children in the TDC group are clustered towards the bottom. While it is the case that group differences leading to clustered data can drive spurious correlations [78], we argue that this is only an issue when it results in a bimodal distribution for one or both of the variables being correlated. Given that SEQ hyper- and hyporeactivity, SEQ sensory seeking, SM1 Glx and Thal GABA (i.e., the variables in the associations presented in Fig. 4a–c) are not bimodally distributed and are approximately normal (see Supplementary Figs. 3 and 4), we believe these associations reflect true associations between hyper- and hyporeactivity with SM1 Glx levels. That said, existence of a clear sub-cluster of those with ASD with high Glx that show high SEQ scores could be indicative of a specific phenotype with potential clear biological markers. There were no associations between Thal Glx with any of the total scores from the SEQ (all *p*_Bonferroni_ > 0.999). There were also no moderating effects of Group (all *p*_Bonferroni_ > 0.999).

#### Thal GABA+ is associated with sensory seeking behaviors

There were no significant associations between SM1 GABA+ and any of the total scores on the SEQ (all *p*_Bonferroni_ > 0.060*)*. There was also no significant moderating effect of Group (all *p*_Bonferroni_ > 0.969).

There was a trend towards an association between Thal GABA+ and parent reported sensory seeking total scores (*r* = 0.34, *p*_Bonferroni_ = 0.054; Fig. 4c). There was otherwise no meaningful association between Thal GABA+ with either hyper- (*r* = 0.21, *p*_Bonferroni_ = 0.234) or hyporeactivity (*r* = 0.17, *p*_Bonferroni_ = 0.750). There was also no meaningful or significant moderating effect of Group (all *p*_Bonferroni_ > 0.244).

#### Thal GABA+ is associated with ADOS communication scores

We also explored whether there were any associations between autism severity and individual differences in metabolite concentration for each region. We identified an association between Thal GABA (when referenced to both water and creatine) and ADOS communication scores (*r* = 0.44, *p*_Bonferroni_ = 0.081, GABA+/Cr: *r* = 0.54, *p*_Bonferroni_ = 0.022), which we highlight in the Supplementary Fig. 5.

### Correlations between metabolite levels of SM1 and Thal with tactile sensitivity

#### SM1 and Thal Glx with tactile perception

When collapsing across groups, there was evidence for linear associations between SM1 Glx levels and sequential frequency discrimination thresholds (*r* = −0.22, *p*_Bonferroni_ = 0.158; Fig. 4d) and the effect of simultaneity (i.e., the difference between simultaneous and sequential frequency discrimination thresholds; *r* = 0.27, *p*_Bonferroni_ = 0.052), though these associations did not survive correction for multiple comparisons. There were no significant moderating effects of Group for any of the associations between SM1 Glx and tactile sensitivity (all *p*_Bonferroni_ > 0.885).

For Thal Glx levels, group collapsed correlation analyses found an association between Thal Glx and simultaneous frequency discrimination thresholds (*r* = 0.31, *p*_Bonferroni_ = 0.164; Fig. 4e), though this association also did not survive correction for multiple comparisons. Interestingly, however, there was some evidence for a moderating effect of Group on the association between Thal Glx and feedforward inhibition [*F*(1, 40) = 7.35, *p*_Bonferroni_ = 0.040, BF_10_ = 0.349]. Simple slope analyses revealed that while Thal Glx showed no association to feedforward inhibition in TDC (*r* = 0.14, *p*_Bonferroni_ = 0.960), there was a strong negative association between Thal Glx and feedforward inhibition in ASD (*r* = −0.63, *p*_Bonferroni_ = 0.018; Fig. 4f).

#### No association between SM1 and Thal GABA+ with tactile perception

There were no significant associations between SM1 GABA+ levels with any of the tactile perceptual thresholds (all *p*_Bonferroni_ > 0.224), nor was there evidence of a moderating effect of Group for any of the associations (all *p*_Bonferroni_ > 0.999). Like SM1 GABA+ , there were no significant associations between Thal GABA+ levels with any of the tactile perceptual thresholds (all *p*_Bonferroni_ > 0.960), nor was there evidence of a moderating effect of Group (all *p*_Bonferroni_ > 0.999).

## Discussion

In contrast to previous work showing altered GABA+ in ASD [29, 30, 42, 79, 80], and in contrary to our hypotheses, GABA+ levels in both SM1 and Thal were comparable between children in the ASD and TDC groups. Instead, when compared to TDCs, Glx levels were significantly higher in the SM1 of the ASD group. Indeed, this finding could be interpreted as supporting the E-I balance theory of sensory symptoms in ASD (i.e., by increased ‘excitation’). These results are also consistent with previous studies finding altered Glx levels in ASD [81–90]. We also identified a strong positive association between SM1 and Thal Glx in the ASD group that was otherwise absent in the TDC group, supporting the well-replicated and robust findings of hyperconnectivity between thalamic and sensory cortical regions in ASD [91–95].

While altered E-I balance has long been suggested to underlie the sensory symptoms of ASD, clear links between markers of excitation and inhibition to sensory symptoms of ASD were scarce. We took a comprehensive approach to linking excitation and inhibition to sensory symptoms, measuring both GABA+ and Glx levels as markers of E-I, as well as assessing sensory symptoms at both behavioral and perceptual levels. Behaviorally, our results revealed that children with higher Glx levels in SM1 were also those who were reported as being more hyper- and hyporeactive to sensory input. At the perceptual level, both SM1 and Thal Glx levels were loosely associated to differences in low-level tactile discrimination. Perhaps most strikingly, our results also revealed that children with ASD who had higher levels of Thal Glx also showed less feed-forward inhibition, a process known to be critical for preventing over-excitation and reducing task-irrelevant noise during signal processing [96].

When taken in the context of our previous work which had highlighted an association between low-level perceptual alterations and the symptoms of sensory hyper- and hyporeactivity in ASD [16], we can begin to construct a model of sensory abnormalities in ASD. First, elevated Glx levels, which is indirectly indicative of increased glutamatergic signaling, may explain greater difficulties with sensory discrimination and more difficulties with sensory gating. Due to these difficulties with discrimination and feedforward inhibition, individuals with ASD may experience the world as being noisier than their neurotypical counterparts. In turn, this may explain why individuals with ASD tend to be both hyper- and hyporeactive to sensory input. Indeed, noisier environments can be conducive to both greater levels of discomfort, as well as more difficulties with detecting relevant signals. Discomfort with the sensory environment can understandably result in hyperreactive responses to sensory input and difficulties with detecting relevant signals could feasibly explain hyporeactive responses since the lack of detection of relevant signals amongst noise could result in the absence of an otherwise expected response.

As always, we must consider these results in the context of the limitations of the study and its methods. First, we recognize that the ASD sample in the current study generally consist of individuals with ‘high-functioning’ autism. Moreover, the total sample consists of a narrow age range (by design) and is also predominantly male. These limitations do affect the generalizability of our findings. Next, while the E-I balance theory of ASD considers excitatory glutamatergic and inhibitory GABAergic signaling as independent processes, with regard to our MRS markers of both glutamate and GABA+ , the picture is more complicated. As we have already discussed, our measures of glutamate are contaminated with glutamine. Amongst other roles [97–99], glutamine also functions as a precursor for the biosynthesis of GABA via glutamate [100], meaning that some of the Glx being measured represents glutamine and glutamate that function as precursors to GABA. Thus, while we consider measures of Glx and GABA+ as independent markers of excitation and inhibition respectively, MRS measures of Glx and GABA+ are interrelated. Moreover, and perhaps more critically, concentration levels do not directly reflect glutamatergic and GABAergic neurotransmission, but rather relate to total metabolite pools that certainly contribute to excitation and inhibition, but also have clear roles in energy and nitrogen metabolism [101]. Thus, while these pools contribute to E-I, they might reflect more general disruptions of Glu-GABA metabolism. Considering the importance of these limitations, we provide additional discussion of MRS-related methodological implication and how they might explain the discrepancies between the current and previous findings regarding GABA+ in the Supplementary Discussion.

In summary, the present study identified region-specific elevations of Glx in the SM1 of children with ASD. Individual differences in SM1 Glx levels were significantly associated with parent reports of hyper- and hyporeactivity, as well as psychophysically derived measures of perceptual sensitivity in the tactile domain. Collectively, our findings provide strong evidence in support of the altered E-I balance theory of ASD. The results suggest that alterations at the neurobiological implementation of glutamatergic signaling may be related to alterations of tactile perception, which in turn may explain the observation of hyper- and hyporeactivity in ASD.

## Supplementary information

Supplementary Material
