# Supplementary material for: Region-specific elevations of glutamate + glutamine correlate with the sensory symptoms of autism spectrum disorders
Source: Transl Psychiatry. 2021 Jul 29;11:411. doi: 10.1038/s41398-021-01525-1 (PMC8322079; doi:10.1038/s41398-021-01525-1)
Supplement: Supplementary file 1 — Supplementary Material [file 41398_2021_1525_MOESM1_ESM.docx]

**Supplementary Materials: Region-specific elevations of glutamate + glutamine correlate with the sensory symptoms of autism spectrum disorders**

Jason L. He^1,2,3,*^, Georg Oeltzschner^1,2,*^, Mark Mikkelsen^1,2^, Alyssa Deronda^4^, Ashley D. Harris^5^, Deana Crocetti^4^, Ericka L. Wodka^6^, Stewart H. Mostofsky^4,7,8^, Richard A. E. Edden^1,2^, Nicolaas A. J. Puts^1,2,3,9^

^1^*Russell H. Morgan Department of Radiology and Radiological Science, The Johns Hopkins University School of Medicine, Baltimore, MD, United States*

*^2^F. M. Kirby Research Center for Functional Brain Imaging, Kennedy Krieger Institute, Baltimore, MD, United States*

*^3^Department of Forensic and Neurodevelopmental Sciences, Sackler Institute for Translational Neurodevelopment, Institute of Psychiatry, Psychology, and Neuroscience, King's College London, London, UK*

*^4^Center for Neurodevelopmental and Imaging Research, Kennedy Krieger Institute, Baltimore, MD, United States*

*^5^ Department of Radiology, University of Calgary, Canada*

*^6^* *Center for Autism and Related Disorders, Kennedy Krieger Institute, Baltimore, MD United States*

*^7^Department of Neurology, The Johns Hopkins University School of Medicine, Baltimore, MD, United States.*

^8^ *Department of Psychiatry and Behavioral Sciences, The Johns Hopkins University School of Medicine, Baltimore, MD, United States.*

^9^ *MRC Centre for Neurodevelopmental Disorders, King’s College London*

*Authors contributed equally to the manuscript

**Summary**

The work contained in this document are provided as supplementary to the main manuscript. The Supplemental Methods further detail the MRS acquisition, pre-processing, modelling and quantification. In addition, the Supplemental Methods also contain an in-depth explanation of the tactile psychophysical battery used to test tactile perceptual thresholds and descriptive tables of the metabolite data (and relevant quality assurance metrics). The supplemental results are parallel to the ones presented in the main manuscript, with the only exception being that the reference variable is creatine rather than water. These parallel analyses are intended to assess whether the effects we identified with our water referenced metabolites (i.e., Glx (IU) and GABA (IU)).

**Supplemental Methods**

**Comprehensive detailing of MRS data acquisition, pre-processing, modelling and quantification.**

**Acquisition.** Structural and MRS data were acquired between February 2014 and December 2019 on a Philips 3T MRI scanner (Philips Healthcare, Best, The Netherlands). For all scans, a 32-channel phased-array head coil was used for receiving, and the body coil for transmitting. Following a fast survey image, a high-resolution (1 mm^3^ isotropic) *T*_1_-weighted (MPRAGE) image was acquired to guide voxel placement and to be used for tissue segmentation during data analysis.

During the acquisition period, the MRI scanner underwent hardware and software upgrades. In addition, our methodological research in the field of edited MRS revealed that the initially chosen MRS protocol (macromolecule-suppressed MEGA-PRESS to edit for more ‘pure’ GABA measurement^1,2^) was extremely vulnerable to gradient-induced frequency drift and editing pulse. Upon recognizing these disadvantages (described further below in the Supplemental Discussion), our group developed several technical improvements that address and mitigate the deleterious effects of drift and frequency offsets^3–5^. Later, the new acquisition technique HERMES^3^ became available, allowing for simultaneous spectral editing of GABA and glutathione (GSH) with a single multiplexed editing scheme, which was adopted. The different parameters for each phase were:

- **Phase 1:** Macromolecule-suppressed GABA-edited MEGA-PRESS^6,7^ (February 2014 - April 2015); 20-ms editing pulses applied in alternating fashion at 1.9 ppm (GABA-ON) and 1.5 ppm (GABA-OFF); separate water-unsuppressed reference scan (TE = 80 ms) after the water-suppressed scan.
- **Phase 2:** Macromolecule-suppressed GABA-edited MEGA-PRESS (August 2016 - July 2018) with interleaved water referencing and crusher-gradient lock; 20-ms editing pulses applied in alternating fashion at 1.9 ppm (GABA-ON) and 1.5 ppm (GABA-OFF); interleaved water-unsuppressed reference scan (TE = 80 ms) and dynamic scanner frequency update every 20 averages^8^ crusher-gradient lock to prevent rotation-dependent eddy-current-induced editing pulse frequency offset^9^.
- **Phase 3:** HERMES^3^ for simultaneous editing of GABA + macromolecules (GABA+) and GSH (July 2018 - December 2019). 20-ms editing pulses applied in a four-step scheme: (A) 1.9 ppm & 4.56 ppm, (B) 1.9 ppm, (C) 4.56 ppm, (D) no editing pulse; interleaved water-unsuppressed reference scan (TE = 80 ms) and dynamic scanner frequency update every 20 averages.

In all phases, an isotropic MRS voxel (30 × 30 × 30 mm^3^ [27 ml]) was placed in the right sensorimotor cortex (SM1), since tactile stimulation was performed on the left hand. The voxel was centered using the hand knob in the central sulcus as a guiding anatomical landmark^10^, and rotated to be aligned with the dorsolateral surface (Figure 2a). For phases 2 and 3, an additional MRS voxel (26 mm (AP) × 24 mm (CC) × 40 mm^3^ (LR) [25 ml]) was placed in the thalamus, spanning both hemispheres (Thal). This voxel was positioned to include both halves of the thalamus, sacrificing information on laterality to achieve sufficient signal-to-noise ratio from this deep-lying brain region.

**Pre-processing, modelling, and quantification.** All MRS data and structural images were processed using Gannet (version 3.1), a software package optimized for the processing of edited MRS data^11^. Spectral pre-processing (performed by the GannetLoad module) included 3-Hz exponential line broadening, zero-filling to 32768 points, frequency-and-phase correction of individual averages using the spectral registration method^12^, averaging, and subtraction of the edited subspectra to yield GABA-edited (and, for HERMES data, GSH-edited) difference spectra.

The GannetFit module was then used to model the difference spectra, edit-OFF spectra, and water spectra. In GABA-edited difference spectra, a combined GABA-Glx model between 2.79 ppm and 4.1 ppm was used to fit the 3-ppm GABA resonance and the 3.75-ppm Glx doublet simultaneously. This model includes a single Gaussian signal for the GABA peak (between 2.79 ppm and 3.2 ppm), a double-Gaussian peak for the Glx double (between 3.4 ppm and 4.1 ppm), as well as linear, cosine, and sine baseline terms to account for baseline distortions resulting from residual water or lipid signals. In edit-OFF spectra, the prominent singlets from total N-Acetylaspartic Acid (NAA; 2.05 ppm), creatine (at 3.02 ppm) and total choline (3.20 ppm) were fit with a double-Lorentzian model between 2.6 ppm and 3.6 ppm, with a fixed peak separation (0.18 ppm), a zero-order phase term, and a linear baseline term. Water spectra were fit between 3.8 ppm and 5.6 ppm using a single mixed Lorentzian-Gaussian model including a linear baseline term and a vertical offset.

Using the GannetCoregister and GannetSegment modules, each voxel was co-registered to the *T*_1_-weighted anatomical image from the same acquisition. Using tissue segmentation implemented in SPM12, the fractional tissue volumes for grey matter (GM), white matter (WM), and cerebrospinal fluid (CSF) were determined for each voxel. The model areas for the different metabolites and the segmentation results were then used to derive different quantitative estimates for each metabolite: 1) creatine ratios *GABA/Cr* and *Glx/Cr* (without any further correction); 2) tissue-, relaxation-, and alpha-corrected concentration estimates *GABAAlphaCorr and GlxAlphaCorr (in Institutional Units, IU)* were derived as published previously^13^, which accounts for the different amount of GABA between grey and white matter, assuming a 2:1 ratio (i.e., grey matter GABA levels are twice as high as white matter GABA levels). Although the alpha for Glx is less well-known, here we assume a similar alpha as for GABA as per previously suggested^14–16^. The final quantitative metabolite estimates were then passed on to the statistical analysis. Taking metabolite estimates relative to total creatine and tissue-water into account helps establish that potential group effects and correlations are indeed driven by individual differences in the nominator (GABA or Glx, respectively), rather than in the denominator (water or creatine, respectively).

The GannetLoad and GannetFit output plots for each dataset were visually inspected by a member of the study team (GO, ~9 years of experience using edited MRS). Individual fits were excluded from further analyses if the data themselves or the fits were considered unusable (for example, due to excessive subject motion, resulting in strongly diminished spectral quality, e.g. subtraction artefacts and lipid contamination in cortical voxels).

**Table 1. Descriptive statistics for Glx and GABA+ across acquisition phases for both SM1 and Thal**

|  | ASD | | | TDC | | |  |  |
| --- | --- | --- | --- | --- | --- | --- | --- | --- |
| Variable | N | M | SD | N | M | SD | Phase | Region |
| Glx/Cr | 1 | 0.14 | NA | 11 | 0.08 | 0.03 | Phase 1 | SM1 |
| Glx (IU) | 1 | 15 | NA | 11 | 9.22 | 2.63 | Phase 1 | SM1 |
| GABA+/Cr | 1 | 0.15 | NA | 11 | 0.1 | 0.05 | Phase 1 | SM1 |
| GABA+ (IU) | 1 | 4.79 | NA | 11 | 3.11 | 1.34 | Phase 1 | SM1 |
| Glx/Cr | 19 | 0.03 | 0.01 | 22 | 0.03 | 0 | Phase 2 | SM1 |
| Glx (IU) | 19 | 3.02 | 0.51 | 22 | 3.16 | 0.22 | Phase 2 | SM1 |
| GABA+/Cr | 19 | 0.07 | 0.01 | 22 | 0.08 | 0.01 | Phase 2 | SM1 |
| GABA+ (IU) | 19 | 2.14 | 0.23 | 22 | 2.22 | 0.26 | Phase 2 | SM1 |
| Glx/Cr | 24 | 0.08 | 0.01 | 26 | 0.07 | 0.02 | Phase 3 | SM1 |
| Glx (IU) | 24 | 8.53 | 1.7 | 26 | 6.4 | 1.81 | Phase 3 | SM1 |
| GABA+/Cr | 24 | 0.1 | 0.02 | 26 | 0.1 | 0.01 | Phase 3 | SM1 |
| GABA+ (IU) | 24 | 3.01 | 0.52 | 26 | 2.72 | 0.37 | Phase 3 | SM1 |
| Glx/Cr | 13 | 0.03 | 0 | 24 | 0.03 | 0.01 | Phase 2 | Thal |
| Glx (IU) | 13 | 3.46 | 0.65 | 24 | 3.23 | 0.79 | Phase 2 | Thal |
| GABA+/Cr | 13 | 0.09 | 0.01 | 24 | 0.09 | 0.01 | Phase 2 | Thal |
| GABA+ (IU) | 13 | 2.91 | 0.56 | 24 | 2.96 | 0.42 | Phase 2 | Thal |
| Glx/Cr | 16 | 0.08 | 0.01 | 9 | 0.08 | 0.01 | Phase 3 | Thal |
| Glx (IU) | 16 | 8.8 | 1.46 | 9 | 8.38 | 0.83 | Phase 3 | Thal |
| GABA+/Cr | 16 | 0.12 | 0.03 | 9 | 0.11 | 0.03 | Phase 3 | Thal |
| GABA+ (IU) | 16 | 4.32 | 1.18 | 9 | 3.63 | 0.94 | Phase 3 | Thal |

Glx = glutamate + glutamine, Cr = creatine, NAA = N-Acetylaspartic Acid, IU = institutional units, SM1 = primary sensorimotor cortex, Thal = thalamus, ASD = autism spectrum disorders, TDC = typically developing controls. *Note: see main text for descriptive statistics for residual metabolite estimates.*

**Table 2. Descriptive statistics for quality assurance metrics**

|  |  | All |  |  | ASD |  |  | TDC |  |  |
| --- | --- | --- | --- | --- | --- | --- | --- | --- | --- | --- |
| SM | N | M | SD | N | M | SD | N | M | SD | p |
|  |  |  |  |  |  |  |  |  |  |  |
| Glx/Cr Fit Error | 103 | 4.68 | 1.54 | 44 | 4.78 | 1.8 | 59 | 4.6 | 1.32 | 0.57 |
| Glx (IU) Fit Error | 103 | 4.36 | 1.59 | 44 | 4.49 | 1.87 | 59 | 4.26 | 1.34 | 0.48 |
| Glx signal:noise | 103 | 20.23 | 5.86 | 44 | 20.17 | 6.8 | 59 | 20.28 | 5.11 | 0.93 |
| GABA+/Cr Fit Error | 103 | 5.49 | 1.87 | 44 | 5.65 | 1.68 | 59 | 5.38 | 2.01 | 0.47 |
| GABA+ (IU) Fit Error | 103 | 5.22 | 1.91 | 44 | 5.4 | 1.74 | 59 | 5.09 | 2.04 | 0.4 |
| GABA signal:noise | 103 | 16.95 | 5.05 | 44 | 16.04 | 4.32 | 59 | 17.62 | 5.47 | 0.1 |
| NAA FWHM | 103 | 7.45 | 1.39 | 44 | 7.77 | 1.92 | 59 | 7.2 | 0.73 | 0.07 |
| Frequency Drift | 103 | -0.83 | 0.84 | 44 | -0.73 | 0.84 | 59 | -0.91 | 0.84 | 0.29 |
| Thal |  |  |  |  |  |  |  |  |  |  |
| Glx/Cr Fit Error | 62 | 8.52 | 3.34 | 29 | 7.79 | 2.91 | 33 | 9.17 | 3.59 | 0.1 |
| Glx (IU) Fit Error | 62 | 8.34 | 3.4 | 29 | 7.59 | 2.98 | 33 | 9.01 | 3.64 | 0.1 |
| Glx signal:noise | 62 | 11.29 | 4.93 | 29 | 11.82 | 4.79 | 33 | 10.83 | 5.08 | 0.43 |
| GABA+/Cr Fit Error | 62 | 7.06 | 2.27 | 29 | 6.99 | 2.34 | 33 | 7.12 | 2.25 | 0.83 |
| GABA+ (IU) Fit Error | 62 | 6.86 | 2.3 | 29 | 6.78 | 2.37 | 33 | 6.93 | 2.28 | 0.8 |
| GABA signal:noise | 62 | 12.75 | 2.9 | 29 | 12.56 | 2.77 | 33 | 12.93 | 3.04 | 0.62 |
| NAA FWHM | 62 | 8.8 | 0.93 | 29 | 8.85 | 0.99 | 33 | 8.75 | 0.88 | 0.67 |
| Frequency Drift | 62 | -0.97 | 0.82 | 29 | -0.72 | 0.84 | 33 | -1.18 | 0.75 | 0.03 |

IU = institutional units, SM1 = primary sensorimotor cortex, Thal = thalamus, ASD = autism spectrum disorders, TDC = typically developing controls. *Note: see main text for descriptive statistics for residual metabolite estimates. P-values in the last column reflect group comparisons made using independent samples t-test.*

**Tactile acquisition & processing**

Each participant was tested on a battery of vibrotactile protocols^17^. To complete the battery, participants were required to rest their left hand on a CM4 four-digit tactile stimulator (Cortical Metrics, Carrboro, NC) and their right hand on a standard wired computer mouse. The CM4 delivered flutter range (25 – 50 Hz) stimuli to the glabrous skin of the left index (left digit 2; LD2) and middle finger (left digit 3; LD3) of each participant via two cylindrical plastic probes (diameter: 5mm). The battery consisted of 11 separate tasks grouped into five domains (reaction time, detection threshold, amplitude discrimination, frequency discrimination, and temporal order judgment), with three conditions in the amplitude discrimination domain and two conditions for each of the other domains. A computer running custom Cortical Metrics scripts was used to visualize each trial and control the parameters of the vibrotactile stimuli (i.e., timing, duration, amplitude and frequency) of each trial for each protocol. Data were collected and stored on the same computer. The data were then later processed and visualized using a custom R package (available at: <https://github.com/HeJasonL/BATD>).

To confirm that participants understood the instructions, each task was preceded by three practice trials which had to be answered correctly to proceed to the testing stage. Participants were given additional practice if they were unable to do this in their first attempt. Feedback was only given during practice trials. All responses were obtained via the mouse under the participants’ right hand. The left mouse button corresponded to LD3 and the right mouse button to LD2. For all protocols other than the reaction time and dynamic detection threshold tasks, a stepwise tracking procedure was used to identify participants’ perceptual thresholds in a series of two-alternative forced choice (2-AFC) trials. In protocols in which stepwise tracking was implemented, correct responses made the choice of the subsequent trial more difficult, while incorrect responses made the choice less difficult (described in detail for each of the protocols below). The full battery took approximately 40 minutes to finish for each participant (the full battery contained tasks not described here, but can are described in in our previous publications^17,18^).

**Static detection.** A suprathreshold stimulus (frequency *=* 25 Hz; starting amplitude *=* 20 μm; duration *=* 500 ms) was pseudorandomly delivered to either LD2 or LD3. Participants were asked to respond on which finger they felt the stimulus. A one-up–one-down tracking paradigm (stimulus amplitude was decreased for a correct answer and increased for an incorrect answer) was used for the first 10 trials and a two-up–one-down (two correct answers were necessary for a reduction in test amplitude) was used for the remainder of the task. There were 24 trials. Static detection threshold was calculated as the mean of the amplitudes of the last five trials.

**Dynamic detection.** In the dynamic detection condition, a 25-Hz stimulus increased from zero amplitude at a rate of 2 μm/s. Participants were asked to respond on which finger they felt the stimulus as soon as they felt it. Each trial began with a variable delay (0-2500 ms) and trials were delivered with an inter-trial interval of 10 s. There were 7 trials in the dynamic detection protocol. Dynamic detection thresholds were calculated as the mean stimulus amplitude at the time of pressing the button, across all correct trials. Feed-forward inhibition was then estimated to be dynamic detection threshold divided by static detection threshold multiplied by 100.

**Amplitude discrimination.** Stimuli (frequency *=* 25 Hz; duration *=* 500 ms; a 5-s inter-trial interval) were delivered to LD2 and LD3 simultaneously. One finger always received the standard stimulus (amplitude = 100 μm) while the other received the comparison stimulus (initial amplitude = 200 μm). The two stimuli were delivered to either digit pseudorandomly. Participants were asked to choose which of two simultaneously delivered stimuli had the higher amplitude.

A one-up–one-down tracking paradigm (comparison stimulus amplitude was decreased by 10 μm for a correct answer and increased by 10 μm for a wrong answer) was used for the first 10 trials and a two-up–one-down was used for the remainder of the task (20 trials in total or 20 more trials). Amplitude discrimination thresholds were calculated as the mean of the amplitudes of the last five trials.

**Sequential frequency discrimination.** Frequency discrimination of sequentially delivered stimuli was tested. Stimuli (duration *=* 500 ms; amplitude *=* 200 μm) were delivered to left digit 2 and left digit 3 simultaneously to both digits, separated by a 500ms inter-trial interval (ISI). One finger always received the standard stimulus (frequency *=* 30 Hz) while the other received the comparison stimulus (initial frequency *=* 40 Hz). The two stimuli were delivered to either digit pseudo-randomly (20 trials with an inter-trial interval = 5s). Participants were asked which finger received the higher frequency stimulus.

A one-up–one-down tracking paradigm (the comparison stimulus frequency was decreased for a correct answer and increased for a wrong answer) was used for the first 10 trials. A two-up–one-down was used for the remainder of the trials. Frequency discrimination thresholds were obtained as the mean of the frequencies of the last five trials.

**Simultaneous Frequency discrimination.** The simultaneous frequency discrimination protocol was identical to the sequential; frequency discrimination protocol with the exception that the stimuli were delivered simultaneously rather than sequentially. The effect of simultaneity was estimated as simultaneous discrimination threshold divided by sequential discrimination threshold multiplied by 100.

**Table 3. Descriptive statistics for performance on the tactile protocols**

|  | ASD | | | TDC | | |
| --- | --- | --- | --- | --- | --- | --- |
| Variable | N | M | SD | N | M | SD |
| Static detection | 42 | 11.02 | 5.52 | 61 | 7.90 | 3.65 |
| Dynamic detection | 39 | 9.53 | 5.42 | 59 | 8.96 | 5.26 |
| Feed-forward inhibition | 40 | 15.55 | 89.22 | 60 | 35.14 | 77.45 |
| Amplitude discrimination | 42 | 68.02 | 37.06 | 51 | 58.71 | 33.63 |
| Frequency discrimination | 38 | 41.29 | 4.45 | 59 | 40.13 | 4.89 |
| Sequential frequency discrimination | 35 | 40.28 | 4.02 | 58 | 39.44 | 4.31 |
| Simultaneous - Sequential FD | 33 | 4.85 | 11.78 | 57 | 2.36 | 13.14 |

ASD = autism spectrum disorders, TDC = typically developing controls, FD = frequency discrimination

**Supplemental Results**

The analyses described below were conducted to accompany the analyses we conducted on the metabolites referenced to water (i.e., Glx (IU) and GABA+ (IU)). Group means and standard deviations are presented in the main manuscript and visualised in the accompanying figures.

**Comparing metabolite levels of SM1 and Thal between ASD and TDCs.**

**Elevated levels of Glx/Cr in SM1 but not Thal in ASD.** While there was evidence for a significant main effect of Group on Glx/Cr levels [F(1, 145) = 9.11, p = 0.003; η_p_^2^ = 0.06; BF_10_ = 10.42], there was also evidence for a Group by Region interaction effect [F(1, 146) = 3.14, p = 0.078; η_p_^2^ = 0.02; BF_10_ = 1.92]. See Fig. 1a. Subsequent simple main effect analyses found strong evidence for an effect of Group on Glx/Cr levels of the SM1 voxel [F(1,101) = 9.02, p = 0.003; η_p_^2^ = 0.082; BF_10_ = 10.51] which was otherwise absent for the Thal voxel [F(1, 44) = 0.23, p = 0.636; η_p_^2^ = 0.005; BF_10_ = 0.32]. See Fig. 1b. The pattern of results here parallels the water referenced results presented in the main manuscript.

**Comparable levels of GABA+/Cr in SM1 and Thal between ASD and TDCs.** In comparison to Glx/Cr, there was evidence against a main effect of both Group [F(1,146) = 0.01, p = 0.913, η_p_^2^ = 0.000; BF_10_ = 0.18] and Region [F(1, 146) = 0.00, p = 0.999; η_p_^2^ = 0.00; BF_10_ = 0.18] for GABA+ /Cr levels. There was also evidence against an interaction effect [F(1, 146) = 0.01, p = 0.907; η_p_^2^ = 0.000; BF_10_ = 0.03]. See Fig. 1d. Thus, like the results with Glx, these results parallel those presented in the main manuscript.

**Between region correlation of metabolites: evidence for increased glutamatergic thalamocortical connectivity in ASD.** While we identified a positive correlation between SM1 Glx/Cr and Thal Glx/Cr at the whole sample level [(r = 0.29, F(1, 54) = 4.92, p = 0.031; η_p_^2^ = 0.084; BF_10_ = 2.00)], there was evidence towards a moderating effect of Group [(F(1, 52) = 8.36, p = 0.006); η_p_^2^ = 0.14; BF_10_ = 2.36]. Indeed, subsequent simple slope analyses found a non-significant association between SM1 Glx/Cr and Thal Glx/Cr in the TDC group (r = 0.36, p = 0.053), there was a moderate to strong positive correlation in the ASD group (r = 0.4, p = 0.020). See Fig. 1a. While these effects identified with Glx/Cr are weaker than those with Glx (IU), the bulk of the evidence (considering metabolites referenced to both Cr and water) would support no association in TDCs, but a positive association in ASD. For completion, we also assessed inter-region associations of GABA+/Cr. There was no correlation between GABA+/Cr of SM1 and Thal [(r = -0.07, F(1, 54) = 0.24, p = 0.625; η_p_^2^ = 0.004; BF_10_ = 0.30)]. There was also no moderating effect of Group on the associations between SM1 and Thal GABA+/Cr [F(1, 52) = 0.04, p = 0.844; η_p_^2^ = 0.000; BF_10_ = 0.09)]. See Fig. 1b.

**Figure 1. Region-specific increase in Glx/Cr but not GABA+/Cr in ASD a.** Evidence towards a Group by Region interaction effect on Glx/Cr **b.** Higher Glx/Cr levels in the SM1 voxel for children in the ASD compared to TDC group. **c**. Low evidence towards a meaningful group difference in Glx/Cr levels in the Thal voxel**. d**. Lack of evidence towards a significant Group by Region interaction effect on GABA+/Cr levels **e.** Comparable GABA+/Cr in SM1 and **f.** Thal voxels between children in the ASD and TDC groups. **g.** Linear relationship between SM1 Glx/Cr and Thal Glx/Cr in ASD that is otherwise absent in TDC. **h**. No relationship between SM1 GABA and Thal GABA in either ASD or TDC. IU = institutional units, SM1 = primary sensorimotor cortex, Thal = thalamus, ASD = autism spectrum disorders, TDC = typically developing controls. Error bars in panels a and d represent standard error. The shaded area around the line of best fit represents the 95% confidence interval.

**Correlations between metabolite levels of SM1 and Thal with sensory ‘reactivity’**

**SM1 Glx/Cr is associated with hypo- and hyper-reactivity.** There were significant associations between SM1 Glx/Cr and parent/caretaker reported hyper- (r = 0.31, p = 0.008) and hypo-reactivity (r = 0.24, p = 0.041), but not sensory seeking (r = 0.06, p = 0.620) total scores of the SEQ. There were no moderating effects of Group for any of these associations (all p > 0.334). There were no associations between Thal Glx/Cr and any of the total scores from the SEQ (all p > 0.563). There were also no moderating effects of Group on any of those associations (all p > 0.298).

**SM1 GABA+/Cr is also associated with hypo- and hyperreactivity, while Thal GABA+/Cr is associated with sensory seeking.** There was no evidence of any associations between SM1 GABA+/Cr and parent/caretaker reported hyper- (r = 0.05, p = 0.670), hypo-reactivity(r = 0.064, p = 0.600) or sensory seeking (r = 0.15, p = 0.22). There was also no evidence of a moderating effect of Group on these associations (all p > 0.145). There were no associations between Thal GABA+/Cr and any of the SEQ measures (all p > 0.198). There was no significant moderating effects of Group (all p > 0.073).

**Correlations between metabolite levels of SM1 and Thal with tactile ‘sensitivity’**

**SM1 and Thal Glx/Cr with tactile perception.** When collapsing across groups, there was some evidence for linear associations between SM1 Glx/Cr levels and simultaneous frequency discrimination thresholds (r = -0.19, p = 0.073) and the effect of simultaneity (r = 0.24, p = 0.026). See Fig. 2d and 2e. There were no other significant correlations between SM1 Glx/Cr levels and tactile perception (all p > 0.490). There was a trend towards a moderating effect of group on the relationship between SM1 Glx/Cr levels and sequential frequency discrimination thresholds (F(1, 86) = 3.86, p = 0.053). Further analysis found that while there was a strong negative correlation between SM1 Glx/Cr levels and sequential frequency discrimination thresholds in ASD (r = -0.47, p = 0.005), there was no significant association in TDCs (r = 0.14, p = 0.320). There was little evidence for any further moderating effects of Group (all p > 0.212).

There was a positive correlation between Thal Glx/Cr levels and simultaneous frequency discrimination thresholds (r = 0.41, p = 0.006). See Fig. 2d. There were otherwise no significant associations between Thal Glx/Cr and tactile sensitivity (all p > 0.130). While there was no significant moderating effect of Group on the associations between Thal Glx/Cr level and tactile sensitivity (all p > 0.155), the moderating effect of Group on the association between Thal Glx/Cr levels and feedforward inhibition was the effect that closest to statistical significance (p = 0.155) and analysis of the slopes showed a rather similar pattern of effect (negative association in ASD that was absent in TDCs – see Fig. 2f), allowing us to feel confident that the results were not mediated by the reference signal.

**SM1 and Thal GABA+/Cr with tactile perception.** There were no significant associations between SM1 GABA+/Cr levels with any of the tactile perceptual thresholds (all p > 0.184), nor was there evidence of a moderating effect of Group for any of the associations (all p > 0.122). Like SM1 GABA+/Cr, there were no significant associations between Thal GABA+/Cr levels with any of the tactile perceptual thresholds (all p > 0.359), nor was there evidence of a moderating effect of Group for any of the associations (all p > 0.414).

**Figure 2. Associations between Glx/Cr and GABA+/Cr of SM1 and Thal with sensory reactivity and tactile sensitivity.** Positive correlations were identified between Glx/Cr levels of SM1 and **a.** Hyper- and **b.** hyporeactivity scores on the SEQ. **c.** Thal GABA/Cr levels were positively correlated with sensory seeking scores, though the effect was not significant as they were for GABA (IU). It is difficult to determine whether this is due to the reference variable, since both associations are of similar direction and effect size. While **d.** SM1 Glx/Cr levels showed evidence of a negative association to SQFD thresholds, Thal Glx/Cr levels showed evidence of a positive association to SMFD thresholds (**e.**)**. f.** There was a negative association between Thal Glx/Cr levels and feedforward inhibition in the ASD group that was otherwise absent in the TDC group. We note that while these effects are not significant (in comparison to those presented in the main manuscript), the effects are strikingly similar: an interaction effect followed by a negative association in the ASD group that is otherwise absent in the TDC group. IU = institutional units, Glx = glutamate + glutamine, Cr = creatine, SM1 = primary sensorimotor cortex, Thal = thalamus, ASD = autism spectrum disorders, TDC = typically developing controls, SEQ = Sensory Experience Questionnaire, SQFD = sequential frequency discrimination, SMFD = simultaneous frequency discrimination. The shaded area around the line of best fit represents the 95% confidence interval.

**Evidence of non-bimodality of distributions for variables presented in Figure 4**


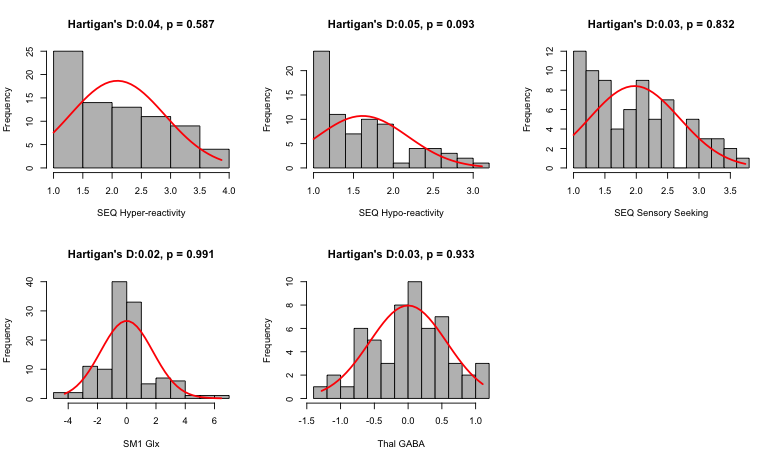


**Figure 3. Evidence of ‘non-bimodal’ density distributions for the variables presented in figures 4a, 4b and 4c of the main manuscript.** Histograms (in grey) and density distribution (red line) of each of the variables in Fig. 4. SEQ = Sensory Experience Questionnaire. D = dip test

*
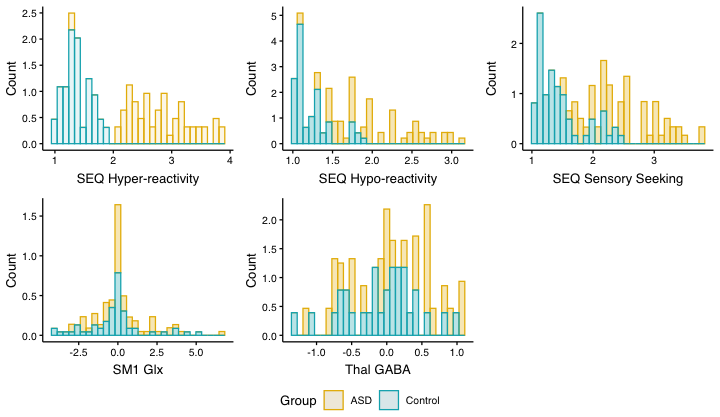
*

**Figure 4. Further evidence of non-bimodal data using overlayed histograms.** To further investigate whether the data were bimodal, we split the groups and plotted the histograms of each of the variables presented in Fig. 4. As Can be discerned, although groups means are different (as was expected given the significant group differences), the modes are approximately the same suggesting these data stem from the same distribution, perhaps with the exception of SEQ Sensory Seeking variable. SM1 = primary sensorimotor cortex, Thal = thalamus, ASD = autism spectrum disorders, TDC = typically developing controls, SEQ = Sensory Experience Questionnaire

**Associations between Glx/Cr and GABA+/Cr of SM1 and Thal with ADOS scores.** We conducted a set of additional exploratory analyses to see whether individual differences in Glx/Cr and GABA+/Cr in SM1 and Thal were associated with autism severity as measured by the ADOS. The only associations we identified that were meaningful, and would have survived multiple corrections, were associations between Thal GABA+/Cr and Thal GABA+/Cr with ADOS communication scores. See Supplementary Fig. 3.

**Figure 5. Associations between Thal GABA+ (IU) and GABA+/Cr with ADOS Communication scores.** Associations were identified regardless of whether Thal GABA+ was referenced to water (**a**) or creatine (**b**). IU = institutional units, Glx = glutamate + glutamine, Cr = creatine, SM1 = primary sensorimotor cortex, Thal = thalamus, ADOS = Autism Diagnostic Observation Scale. The shaded area around the line of best fit represents the 95% confidence interval.

**Supplemental Discussion**

**Data acquisition effects**

Our work is limited by using different acquisition approaches, which is typical for studies with long duration. Sites may change scanner, or software, which may impact sequence performance. Here, initially, a ‘symmetric’ editing pulse scheme to suppress the contribution from co-edited macromolecular signal was chosen for this study in order to obtain a purer measure of GABA than using the more common approach for GABA+ editing. This specific macromolecule-suppressed experiment has since been shown to be extremely sensitive to even small editing pulse frequency offsets, which can be induced by gradient-intensive imaging, subject motion, or uncorrected eddy current effects^8,19^ In practice, the intended benefit of a supposedly purer GABA measure is countered by the increased vulnerability to experimental instabilities (i.e., an unstable scanner). If the symmetry of macromolecule suppression is incomplete, macromolecule signals may contribute positively or negatively to the 3 ppm signal, depending on the individual frequency history of the MRS scan, and add variance to individual GABA estimates. The susceptibility to drift was mitigated by the introduction of a periodic scanner frequency update throughout the macromolecule-suppressed acquisition, but the experiment itself remains fragile, particularly compared to the HERMES^4^ method that was used in the third acquisition phase of the study. HERMES does not employ macromolecule suppression and therefore returns a composite GABA+MM signal that is consistently contaminated with co-edited macromolecules, however, macromolecule-unsuppressed estimates are more robust to small frequency offsets.

Taken together, the measured 3-ppm signals arising from the three phases contain systematically different amounts of co-edited MM signals. While they all represent an index of the amount of GABA molecules in the MRS voxel, the relative contribution of GABA to their composite signals is difficult to estimate and harmonize across acquisition phases, although we aimed to account for this variation by pooling data across phases.

We further highlight the importance of reporting GABA relative to both unsuppressed water and creatine to determine whether any changes are indeed robustly driven by GABA. The methodological limitations discussed above may explain why our results did not replicate the previously shown associations between sensorimotor GABA+ levels and tactile task performance in children and adults (though doing this with data from the current study would result in underpowered analyses).

In contrast, the edited 3.75-ppm Glx signal is not confounded by underlying co-edited MM signals Accurate detection of this signal is not dependent on the variaton of the contribution of macromolecules (since the Glx signals are only affected by the 1.9 ppm, but not the 1.5 ppm editing pulse) and Glx measures are therefore less susceptible to individual experimental instabilities. While systematic differences between the three acquisition phases remain (likely due to a systematic editing pulse frequency offset in phase 2), they will experience less influence by the individual scanner frequency history of a single acquisition and should be adequately addressed by the linear-mixed effects model that was used to remove the variance associated with each phase.

**Caution when using GABA and Glx as markers of excitation and inhibition**

MRS measures of GABA and Glx are frequently interpreted as direct proxy measures of inhibition and excitation, respectively. This is clearly an undue simplification - both molecules reside within multiple functional pools, cycle through various metabolic pathways, and fulfil roles beyond the ascribed synaptic inhibition and excitation. Static levels of both metabolites have been reported to correlate with various functional and behavioral metrics, they are interpreted to reflect ‘GABAergic or glutamatergic tones’, i.e. the capability of the macroscopic system to perform inhibitory or excitatory activity. This activity will in turn greatly depend on other microscopic properties that are inaccessible to MRS, but are likely to be altered in ASD, such as local receptor and transporter densities, molecular synthesis or conversion rates, or receptor affinities. Regardless, our finding of increased Glx have a large effect size and appears to impact sensory functioning in children with ASD. This finding remains consistent with the notion of altered E/I imbalance in ASD and show that these differences may exist at the level of GABA and glutamine levels. However, future work should be undertaken to explore the contribution of Glx (and preferably Glu) to the pathophysiology of ASD. If indeed deemed correct, increased Glx function could reflect an important marker for core symptoms of ASD.

**References**

1. Edden, R. A. E., Puts, N. A. J. & Barker, P. B. Macromolecule-suppressed GABA-edited magnetic resonance spectroscopy at 3T. *Magn. Reson. Med.* (2012) doi:10.1002/mrm.24391.

2. Mescher, M., Merkle, H., Kirsch, J., Garwood, M. & Gruetter, R. Simultaneous in vivo spectral editing and water suppression. *NMR Biomed.* (1998) doi:10.1002/(SICI)1099-1492(199810)11:6<266::AID-NBM530>3.0.CO;2-J.

3. Saleh, M. G. *et al.* Simultaneous edited MRS of GABA and glutathione. *Neuroimage* (2016) doi:10.1016/j.neuroimage.2016.07.056.

4. Oeltzschner, G. *et al.* Hadamard editing of glutathione and macromolecule-suppressed GABA. *NMR Biomed.* (2018) doi:10.1002/nbm.3844.

5. Saleh, M. G. *et al.* Simultaneous editing of GABA and glutathione at 7T using semi-LASER localization. *Magn. Reson. Med.* (2018) doi:10.1002/mrm.27044.

6. Edden, R. A. E., Puts, N. A. J. & Barker, P. B. Macromolecule-suppressed GABA-edited magnetic resonance spectroscopy at 3T. *Magn. Reson. Med.* (2012) doi:10.1002/mrm.24391.

7. Harris, A. D., Puts, N. A. J., Barker, P. B. & Edden, R. A. E. Spectral-editing measurements of GABA in the human brain with and without macromolecule suppression. *Magn. Reson. Med.* (2015) doi:10.1002/mrm.25549.

8. Edden, R. A. E. *et al.* Prospective frequency correction for macromolecule-suppressed GABA editing at 3T. *J. Magn. Reson. Imaging* (2016) doi:10.1002/jmri.25304.

9. Oeltzschner, G. *et al.* Dual-volume excitation and parallel reconstruction for J-difference-edited MR spectroscopy. *Magn. Reson. Med.* (2017) doi:10.1002/mrm.26536.

10. Yousry, T. A. *et al.* Localization of the motor hand area to a knob on the precentral gyrus. A new landmark. *Brain* (1997) doi:10.1093/brain/120.1.141.

11. Edden, R. A. E., Puts, N. A. J., Harris, A. D., Barker, P. B. & Evans, C. J. Gannet: A batch-processing tool for the quantitative analysis of gamma-aminobutyric acid-edited MR spectroscopy spectra. *J. Magn. Reson. Imaging* (2014) doi:10.1002/jmri.24478.

12. Near, J. *et al.* Frequency and phase drift correction of magnetic resonance spectroscopy data by spectral registration in the time domain. *Magn. Reson. Med.* (2015) doi:10.1002/mrm.25094.

13. Harris, A. D., Puts, N. A. J. & Edden, R. A. E. Tissue correction for GABA-edited MRS: Considerations of voxel composition, tissue segmentation, and tissue relaxations. *J. Magn. Reson. Imaging* (2015) doi:10.1002/jmri.24903.

14. Tiwari, V., An, Z., Wang, Y. & Choi, C. Distinction of the GABA 2.29 ppm resonance using triple refocusing at 3 T in vivo. *Magn. Reson. Med.* (2018) doi:10.1002/mrm.27142.

15. Srinivasan, R. *et al.* TE-Averaged two-dimensional proton spectroscopic imaging of glutamate at 3 T. *Neuroimage* (2006) doi:10.1016/j.neuroimage.2005.10.048.

16. Ganji, S. K. *et al.* Measurement of regional variation of GABA in the human brain by optimized point-resolved spectroscopy at 7 T in vivo. *NMR Biomed.* (2014) doi:10.1002/nbm.3170.

17. Puts, N. A. J., Edden, R. A. E., Wodka, E. L., Mostofsky, S. H. & Tommerdahl, M. A vibrotactile behavioral battery for investigating somatosensory processing in children and adults. *J. Neurosci. Methods* (2013) doi:10.1016/j.jneumeth.2013.04.012.

18. He, J. L. *et al.* Disorder-specific alterations of tactile sensitivity in neurodevelopmental disorders. *Commun. Biol.* **4**, 97 (2021).

19. Harris, A. D. *et al.* Impact of frequency drift on gamma-aminobutyric acid-edited MR spectroscopy. *Magn. Reson. Med.* (2014) doi:10.1002/mrm.25009.
